# Relationship Between Left Ventricular Ejection Fraction and ICD‐10 Codes Among Patients Hospitalized With Heart Failure

**DOI:** 10.1002/clc.70055

**Published:** 2024-12-16

**Authors:** Ty J. Gluckman, Shih‐Ting Chiu, Deanna Rider, Phil K. Tseng, James O. Mudd, Joshua D. Remick, Craig Granowitz, Amy Carroll, Slaven Sikirica, Mario E. Canonico, Judith Hsia, Marc P. Bonaca

**Affiliations:** ^1^ Center for Cardiovascular Analytics, Research, and Data Science (CARDS), Providence Heart Institute Providence Health System Portland Oregon USA; ^2^ Providence Research Network, Providence Health System Missoula Montana USA; ^3^ University of Arizona College of Medicine Tucson Arizona USA; ^4^ Sacred Heart Medical Center, Providence Health System Spokane Washington USA; ^5^ Lexicon Pharmaceuticals, Inc The Woodlands Texas USA; ^6^ CPC Clinical Research University of Colorado Health Denver Colorado USA

## Abstract

**Introduction:**

While left ventricular ejection fraction (LVEF) represents an important means by which to classify patients with heart failure (HF), relatively little is known about the distribution of LVEFs among patients hospitalized for HF based on their International Classification of Disease (ICD)‐10 code.

**Methods:**

We performed a retrospective cross‐sectional analysis of patients admitted to a large integrated health system within the western US between January 1, 2018 and October 1, 2022 with a principal diagnosis of HF (defined by ICD‐10 codes: I50.2, systolic HF; I50.3, diastolic HF; I50.4, combined systolic and diastolic HF; I11.0, hypertensive heart disease with HF; and I13.0 and I13.2, hypertensive heart disease with HF and chronic kidney disease).

**Results:**

Over nearly 5 years, 61,238 HF hospitalizations occurred, of which 49,772 (81%) had a LVEF available by echocardiography within the preceding 3 months. Whereas most patients hospitalized with systolic HF (*n* = 2220) as well as systolic and diastolic heart failure (*n* = 1582) had an LVEF ≤ 40% (86.2% and 74.8%, respectively), most patients hospitalized with diastolic HF (*n* = 1542) had an LVEF ≥ 50% (94.0%) (Figure). A much greater range of LVEFs were noted for those with hypertensive heart disease with HF (*n* = 18,092) and hypertensive heart disease with HF and CKD (*n* = 26,336) (Figure).

**Conclusion:**

While there was relatively good concordance between LVEF and the ICD‐10 code‐defined HF type for systolic HF, diastolic HF, and systolic and diastolic HF, these codes represent a small subset (~10%) of total HF hospitalizations.

## Graphical abstract

We performed a retrospective analysis of adults discharged with heart failure (HF) from a large healthcare system, seeking to evaluate the relationship between HF type based on left ventricular ejection fraction (LVEF) and assigned HF ICD‐10 codes. Notable heterogeneity in LVEF was observed for the most prevalent HF types (I11.0/I13.0/I13.2).

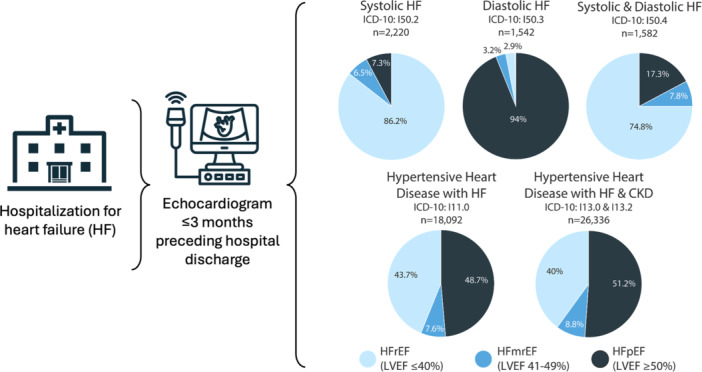

Previous studies have suggested variable performance in how well International Classification of Diseases, Tenth Revision (ICD‐10) diagnosis codes accurately identify specific heart failure (HF) types [1, 2]. In addition, a substantial shift in the distribution of ICD‐10 codes among patients hospitalized with HF was noted in 2017, with hypertensive heart disease with HF and chronic kidney disease (CKD) and hypertensive heart disease with HF becoming the two most common HF diagnoses [3]. Accordingly, we sought to evaluate the relationship between HF type based on left ventricular ejection fraction (LVEF) and assigned HF ICD‐10 codes in a large, contemporary cohort of patients hospitalized for HF.

We performed a retrospective cross‐sectional analysis of adults (≥ 18 years) discharged with HF from a large community‐based healthcare system in the western US (Providence) from January 1, 2018 to October 1, 2022. This was part of a larger effort to better understand the demographics, clinical characteristics, and guideline‐directed medical therapy of patients discharged with HF using an all‐payer data set. HF was defined by ICD‐10 codes assigned as the primary discharge diagnosis (I50.2, systolic HF; I50.3, diastolic HF; I50.4, combined systolic and diastolic HF; I11.0, hypertensive heart disease with HF; I13.0 and I13.2; hypertensive heart disease with HF and CKD).

Patient‐level analyses were not performed; all hospitalizations were considered independent events. The analysis was limited to patients with an available LVEF by echocardiography ≤ 3 months preceding hospital discharge. In cases where > 1 echocardiogram was performed during this period, the echocardiogram closest to discharge was used. The LVEF was used to further categorize the HF type (≤ 40%, HF with reduced ejection fraction [HFrEF]; 41%–49%, HF with mildly reduced ejection fraction [HFmrEF]; ≥ 50%, HF with preserved ejection fraction [HFpEF]). The study was approved by the Providence Institutional Review Board with waiver of informed consent.

A total of 61,238 HF hospitalizations occurred, of which 49,772 (81%) had an LVEF available in the 3 months that preceded hospital discharge. The mean age of patients was 72 years, 46% were female, and 75% were White. Hypertensive heart disease with HF and CKD was the most common ICD‐10 code assigned (*n* = 26,336, 52.9%), followed by hypertensive heart disease with HF (*n* = 18,092, 36.4%), systolic HF (*n* = 2220, 4.5%), combined systolic and diastolic HF (*n* = 1582, 3.2%), and diastolic HF (*n* = 1542, 3.1%). Notable differences in demographic and clinical characteristics were observed across the 5 HF groups, with higher rates of hypertension, hyperlipidemia, diabetes mellitus, and atherosclerotic cardiovascular disease among those with hypertensive heart disease with HF and CKD (data not shown).

Most patients with systolic HF and combined systolic and diastolic HF had an LVEF ≤ 40% (86.2% and 74.8%, respectively) (Figure 1). Similarly, most patients with diastolic HF had an LVEF ≥ 50% (94.0%). In contrast, a much wider mix of LVEFs was noted among patients with hypertensive heart disease with HF (43.7% with an LVEF ≤ 40%, 7.6% with an LVEF of 41–49%, and 48.7% with an LVEF ≥ 50%) and hypertensive heart disease with HF and CKD (40.0% with LVEF ≤ 40%, 8.8% with an LVEF of 41–49%, and 51.2% with an LVEF ≥ 50%).

**Figure 1:**
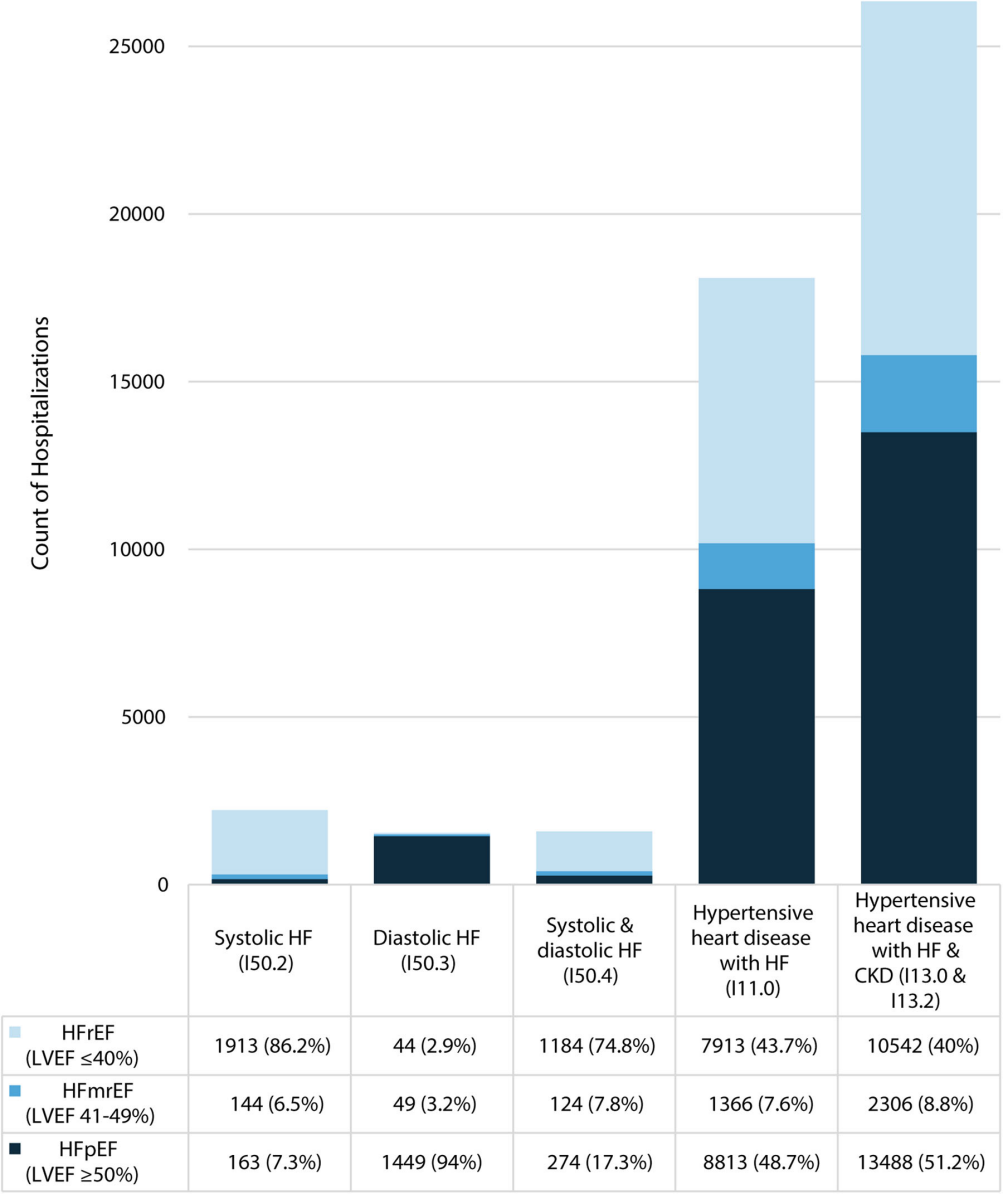
Relationship between discharge ICD‐10 code and type of HF based on LVEF.

The distribution of HF ICD‐10 codes in our cohort closely approximates that observed in a study of fee‐for‐service Medicare beneficiaries after 2017 [3]. Similar to previous studies [2], we also noted a high rate of concordance between assigned diagnoses of systolic HF and an LVEF ≤ 40% and diastolic HF and an LVEF ≥ 50%. Importantly, however, these codes represented < 10% of all HF hospitalizations.

For the 2 largest groups (hypertensive heart disease with HF and CKD and hypertensive heart disease with HF), the combined LVEF distribution and corresponding HF types (41.5% HFrEF, 8.3% HFmrEF, and 50.2% HFpEF) are similar to that noted in a Get With The Guidelines‐HF (GWTG‐HF) registry analysis from 2005 to 2010 [4] and that projected to be present in 2020 [5]. This suggests that while the distribution of assigned ICD‐10 codes has changed appreciably over time, shifts in the specific HF type have been less pronounced.

Our study has several limitations. First, because the cohort was defined exclusively by ICD‐10 codes, it is possible the primary reason for hospitalization was misclassified as HF. Second, patients in our analysis may differ from those hospitalized in other healthcare systems and as such, the findings presented may not be generalizable. Finally, echocardiograms were interpreted locally as opposed to at a core laboratory. As such, interpretive differences may limit the reliability of reported results.

In conclusion, hypertensive heart disease with HF and CKD and hypertensive heart disease with HF were the most common discharge codes, accounting for nearly 90% of HF hospitalizations in a large community‐based healthcare system. While relatively good concordance was noted between LVEF and HF type among those coded as systolic HF, combined systolic and diastolic HF, and diastolic HF, much greater variability in LVEF was noted for those in the other two groups. Accordingly, there is a need to better align coded and clinical data in support of population health and quality improvement efforts to improve HF care.

## Disclosure

Ty J. Gluckman (None), Shih‐Ting Chiu (None), Deanna Rider (None), Phil K. Tseng (None), James O. Mudd (None), Joshua D Remick (Speaker's Bureau: Boehringer Ingelheim), Craig Granowitz (Employee: Lexicon Pharmaceuticals Inc.), Amy Carroll (Employee: Lexicon Pharmaceuticals Inc.), Slaven Sikirica (Employee: Lexicon Pharmaceuticals Inc.), Mario E. Canonico (see below*), Judith Hsia (see below*), Marc P. Bonaca (see below*). *Drs Canonico, Hsia and Bonaca receive salary support from CPC, a non‐profit academic research organization affiliated with the University of Colorado, that receives or has received research grant/consulting funding between July 2021 and July 2023 from the following organizations: Agios Pharmaceuticals Inc., Alexion Pharma Godo Kaisha, Amgen Inc., Anthos Therapeutics Inc., ARCA biopharma Inc., AstraZeneca Pharma India, AstraZeneca Pharmaceuticals LP, AstraZeneca UK Ltd, AstraZeneca Produtos Farmaceuticos, Lda, Atentiv, LLC, Bayer, Bayer (Proprietary) Limited, Bayer Aktiengesellschaft, Bayer Pharma AG, Beth Israel Deaconess Medical Center, Better Therapeutics, Bionest Partners Inc., Boston Clinical Research Institute, LLC, Bristol‐Myers Squibb, CellResearch Corporation Pte Ltd, Cleerly Inc., Colorado Dept of Public Health and Environment, Cook Regentec LLC, CSL Behring LLC, Eidos Therapeutics Inc., EPG Communication Holdings Ltd., Esperion Therapeutics, Inc, Faraday Pharmaceuticals Inc., HeartFlow Inc, Hummingbird Bioscience PTE. LTD., Insmed, Ionis Pharmaceuticals, IQVIA Inc., Janssen Pharmaceuticals, Inc, Janssen Research & Development, LLC, Janssen Scientific Affairs LLC, Lexicon Pharmaceuticals Inc., LSG Corporation, MedImmune Limited, Medpace Inc., Medscape, Merck Sharp & Dohme Corp., Northwell Health, Novartis Pharmaceuticals Corporation, Novo Nordisk, Osiris Therapeutics Inc., Pfizer, PPD Development, L.P., Prothena Biosciences Limited, Regeneron, Regents of the University of Colorado (aka UCD), Sanifit Therapeutics S.A., Sanofi, Silence Therapeutics PLC, Stanford University, Stealth BioTherapeutics Inc., The Brigham & Women's Hospital Inc., Thrombosis Research Institute, UCD iC42 Lab, University of Colorado Denver, University of Pittsburgh, VarmX, WraSer, LLC. Dr. Hsia also reports owning AstraZeneca stock. Dr. Bonaca receives support from the AHA SFRN under award numbers 18SFRN3390085 (BWH‐DH SFRN Center) and 18SFRN33960262 (BWH‐DH Clinical Project). Dr. Bonaca also reports stock in Medtronic and Pfizer.

## Data Availability

The data that support the findings of this study are available from the corresponding author upon reasonable request.

## References

[1] R. J. Desai , M. Mahesri , K. Chin , et al., “Epidemiologic Characterization of Heart Failure With Reduced or Preserved Ejection Fraction Populations Identified Using Medicare Claims,” The American Journal of Medicine 134 (2021): e241–e251. 33127370 10.1016/j.amjmed.2020.09.038

[2] B. A. Bates , E. Akhabue , M. M. Nahass , et al., “Validity of International Classification of Diseases (ICD)‐10 Diagnosis Codes for Identification of Acute Heart Failure Hospitalization and Heat Failure With Reduced Versus Preserved Ejection Fraction in a National Medicare Sample,” Circulation: Cardiovascular Quality and Outcomes 16 (2023): e009078. 36688301 10.1161/CIRCOUTCOMES.122.009078

[3] S. W. Reinhardt , K. A. A. Clark , X. Xin , et al., “Thirty‐Day and 90‐Day Episode of Care Spending Following Heart Failure Hospitalization Among Medicare Beneficiaries,” Circulation: Cardiovascular Quality and Outcomes 15 (2022): e008069. 35861780 10.1161/CIRCOUTCOMES.121.008069

[4] B. A. Steinberg , X. Zhao , P. A. Heidenreich , et al., “Trends in Patients Hospitalized With Heart Failure and Preserved Left Ventricular Ejection Fraction: Prevalence, Therapies, and Outcomes,” Circulation 126 (2012): 65–75. 22615345 10.1161/CIRCULATIONAHA.111.080770

[5] A. A. Oktay , J. D. Rich , and S. J. Shah , “The Emerging Epidemic of Heart Failure With Preserved Ejection Fraction,” Current Heart Failure Reports 10 (2013): 401–410. 24078336 10.1007/s11897-013-0155-7 PMC3870014

